# Genome‐Wide MicroRNA and Gene Analysis of Mesenchymal Stem Cell Chondrogenesis Identifies an Essential Role and Multiple Targets for miR‐140‐5p

**DOI:** 10.1002/stem.2093

**Published:** 2015-07-29

**Authors:** Matt J. Barter, Maria Tselepi, Rodolfo Gómez, Steven Woods, Wang Hui, Graham R. Smith, Daryl P. Shanley, Ian M. Clark, David A. Young

**Affiliations:** ^1^ Institute of Cellular MedicineNewcastle UniversityNewcastle upon TyneUnited Kingdom; ^2^ Institute for Ageing and HealthNewcastle UniversityNewcastle upon TyneUnited Kingdom; ^3^ School of Biological SciencesUniversity of East AngliaNorwichUnited Kingdom

**Keywords:** Mesenchymal stem cells, miRNA, Chondrogenesis, Gene expression, Differentiation, Epigenetics

## Abstract

microRNAs (miRNAs) are abundantly expressed in development where they are critical determinants of cell differentiation and phenotype. Accordingly miRNAs are essential for normal skeletal development and chondrogenesis in particular. However, the question of which miRNAs are specific to the chondrocyte phenotype has not been fully addressed. Using microarray analysis of miRNA expression during mesenchymal stem cell chondrogenic differentiation and detailed examination of the role of essential differentiation factors, such as SOX9, TGF‐β, and the cell condensation phase, we characterize the repertoire of specific miRNAs involved in chondrocyte development, highlighting in particular miR‐140 and miR‐455. Further with the use of mRNA microarray data we integrate miRNA expression and mRNA expression during chondrogenesis to underline the particular importance of miR‐140, especially the ‐5p strand. We provide a detailed identification and validation of direct targets of miR‐140‐5p in both chondrogenesis and adult chondrocytes with the use of microarray and 3′UTR analysis. This emphasizes the diverse array of targets and pathways regulated by miR‐140‐5p. We are also able to confirm previous experimentally identified targets but, additionally, identify a novel positive regulation of the Wnt signaling pathway by miR‐140‐5p. Wnt signaling has a complex role in chondrogenesis and skeletal development and these findings illustrate a previously unidentified role for miR‐140‐5p in regulation of Wnt signaling in these processes. Together these developments further highlight the role of miRNAs during chondrogenesis to improve our understanding of chondrocyte development and guide cartilage tissue engineering. Stem Cells *2015;33:3266–3280*

**Significance Statement**

miRNAs are small genes that have emerged as critical regulators of cell behaviour and differentiation. Here we have used a model of cartilage development from stem cells (chondrogenesis) to identify all miRNA and gene changes during the process. By manipulating this chondrogenesis we are able to define the key miRNAs associated with the cartilage phenotype. Correlating gene and miRNA expression data we identify the particular importance of the ‐5p strand of miR‐140 during chondrogenesis, which we confirm with additional experiments. These developments will enhance our understanding of cartilage tissue engineering, a process with potential as therapy for the treatment of osteoarthritis or focal cartilage defects.

## Introduction

Cartilage is found at the end of bones in articulating joints where it provides both a structural role to resist compression and a lubricating function for low friction movement. Chondrocytes are solely capable of producing cartilage extracellular matrix (ECM) which is largely composed of collagen for structural integrity and proteoglycan for water adsorption. During development chondrocytes also lay the scaffold for long bone formation by terminally differentiating and undergoing hypertrophy to allow resorption and ossification, characteristic of bone formation 1.

In vivo, chondrocytes develop from undifferentiated mesenchymal stem cells (MSCs) in limbs to form cartilage anlage. Cells aggregate to form high density condensations which provide cell‐cell contacts required for initiating the chondrogenic process 1. Essential for this process is the transcription factor SOX9 which in combination with extracellular cues promotes the expression of ECM genes such as the type II collagen characteristic of cartilage 2. This capacity of MSCs is used in vitro, where a similar process driven by TGF‐β leads to formation of a cartilaginous disc in well defined high density cultures or supported scaffold structures 3, 4. Such preparations are potential sources of material in clinical applications to replace damaged or defective cartilage. In particular, sufferers of osteoarthritis (OA), an age‐related degenerative joint disease, experience significant cartilage destruction which might be amenable to replacement by in vitro preparations of autologous MSC‐derived cartilage 5.

Greater understanding of the mechanisms involved in differentiation aids development of viable tissue for tissue engineering applications. microRNAs (miRNAs), which control gene expression post‐transcriptionally, are abundantly expressed in development where they are critical determinants of cell differentiation and phenotype 6. miRNAs are small single‐stranded noncoding RNAs which base‐pair with complementary mRNA targets to elicit transcript repression 7. miRNAs are generally processed from Pol‐II‐derived pri‐miRNA transcripts, initially by Drosha in the nucleus then Dicer cytoplasmically, to form a mature miRNA which is subsequently incorporated into the Argonaute containing RNA‐induced silencing complex (RISC) 8. The miRNA seed sequence, nucleotides 2–7, forms the major mRNA recognition determinant leading to target mRNA translation repression and transcript cleavage by RISC 7. miRNAs are essential for normal skeletal development. Mice with a chondrocyte‐specific deletion of Dicer exhibit severe bone growth defects typified by a reduction in growth plate chondrocyte proliferation and increased hypertrophy 9. A number of miRNAs have been proposed to play important roles in chondrogenesis, in particular miR‐140 and miR‐455 appear to follow a chondrocyte restricted expression profile during development, and miR‐140 is directly regulated by SOX9 10, 11, 12, 13, 14. miR‐140 is upregulated during MSC differentiation into chondrocytes and plays a role in cartilage development and maintenance 11, 15. miR‐140 null mice exhibit a skeletal phenotype with long bone growth retardation again indicative of reduced chondrocyte proliferation 11, 16. Significantly, the miR‐140 null mice also develop articular cartilage damage with age and are more susceptible to surgically induced OA, potentially due to increased Adamts5 levels, a target of miR‐140 11. However, the effect of miR‐140 on Adamts5 is modest and a number of other targets of miR‐140 have been proposed, including Dnpep, Hdac4, IGFBP5, and Smad3 10, 16, 17, 18. As a result, miR‐140 is proposed to modulate a number of cell pathways and functions including platelet‐derived growth factor (PDGF) signaling via the PDGF receptor, and cell proliferation via Sp1 14, 19.

We sought to establish a comprehensive picture of miRNA expression and target regulation in chondrogenesis by integrating miRNA and mRNA expression data. We then further established the requirement for specific differentiation factors, including SOX9, TGF‐β, and cell condensation, in chondrogenesis miRNA expression. This analysis ultimately indicated that the ‐5p strand of miR‐140‐5p is particularly critical in the regulation of chondrocyte gene expression. Consequently we identified and validated a number of novel targets of miR‐140‐5p in both adult articular chondrocytes and during chondrogenesis. This highlighted the diverse range of miR‐140‐5p targets and also indicated a previously unidentified role for miR‐140 in Wnt signaling.

## Materials and Methods

### Human Bone Marrow Stem Cell Culture

Human bone marrow MSCs (from seven donors, 18–25 years of age) were isolated from human bone marrow mononuclear cells (Lonza Biosciences, Berkshire, U.K.) by adherence for more than 24 hours to tissue culture plastic and were expanded in monolayer culture in mesenchymal stem cell growth medium (Lonza Biosciences) supplemented with 5 ng/ml fibroblast growth factor‐2 (R&D Systems, Abingdon, U.K.). Cultures were maintained in a humid atmosphere of 5% CO_2_/95% air at 37°C. Once cells reached confluence, they were passaged (P1) using Trypsin/EDTA at a split ratio of 1:3. Experiments were performed using cells between P2 and P7, and all experiments were repeated with cells from three to four donors. The phenotypes of all donors of MSCs were tested by flow cytometry on a FACSCanto II system (Becton Dickinson, Oxford, U.K.) using a human MSC Phenotyping Kit (Miltenyi Biotec, Bisley, U.K.) with positive staining for CD73, CD90, and CD105 and negative staining for CD14, CD20, CD34, and CD45 (three of which can be found in the supplementary material of reference [20]). Cells were also demonstrated to be capable of differentiation into osteoblastic and adipogenic lineages (Supporting Information Fig. S1 and Supporting Information Materials and Methods).

### Chondrogenic Differentiation

MSCs were resuspended in chondrogenic culture medium consisting of high glucose Dulbecco's modified Eagle's medium containing 100 µg/ml sodium pyruvate (Lonza), 10 ng/ml TGF‐β3 (PeproTech, London, U.K.), 100 nM dexamethasone, 1× ITS‐1 premix, 40 µg/ml proline, and 25 µg/ml ascorbate‐2‐phosphate (all from Sigma‐Aldrich, Poole, U.K.). 5 × 10^5^ MSC in 100 µl medium were pipetted onto 6.5 mm diameter, 0.4‐µm pore size polycarbonate Transwell filters (Merck Millipore, Watford, U.K.), centrifuged in a 24‐well plate (200*g*, 5 minutes), then 0.5 ml of chondrogenic medium was added to the lower well as described 3. Media were replaced every 2 or 3 days up to 14 days.

### Immunohistochemistry and Histology

Transwell discs were fixed in ethanol and processed into paraffin wax. Sections cut at 4 µm were either stained with haematoxylin and eosin or Safranin O/Fast Green (all Sigma), or probed with antibodies raised against collagen type II (Abcam, Cambridge, U.K.). Sections were digested with 1% hyaluronidase for 30 minutes at 37°C and incubated at 4°C overnight with primary antibodies at an appropriate dilution in block solution. After sequential incubation with biotinylated secondary antibody signal was detected with the Vectastain Elite ABC Kit (Vector Laboratories, Peterborough, U.K.) following the manufacturer's instructions and developed using diaminobenzidine (Sigma‐Aldrich). Images were collected using a Zeiss Axioplan 2 (Cambridge, U.K.).

### Chondrocyte Isolation and Culture

Primary human articular chondrocytes (HAC) were derived from articular cartilage obtained from joint replacement patients (from nine donors, 58–80 years of age; Supporting Information Materials and Methods) diagnosed with OA. All tissues were obtained with informed consent and ethics committee approval from the Newcastle and North Tyneside Health Authority. Enzymatic digestion of tissue and maintenance and culture of cells were as previously described 21. When cells reached confluence they were passaged into the appropriate experimentation vessel. Human chondrosarcoma cells (SW1353) were cultured as described 22.

### RNA and miRNA Extraction and Real‐Time Reverse Transcription PCR

Total RNA was isolated from cultured HAC for array expression profiling using Qiagen miRNeasy kit (Qiagen, Crawley, U.K.) following the manufacturer's instructions, and for real‐time RT‐PCR with Ambion Cells‐to‐cDNA II kit (Life Technologies, Paisley, U.K.). Three‐dimensional MSC cartilage discs were disrupted in mirVana miRNA Isolation Kit Phenol (for array expression profiling) or TRIzol (for real‐time RT‐PCR) (both Life Technologies) using a small disposable plastic pestle and an aliquot of Molecular Grinding Resin (G‐Biosciences, St. Louis, MO). Total RNA was converted to cDNA using Invitrogen MMLV reverse transcriptase (Life Technologies) and TaqMan RT‐PCR was performed and gene expression levels calculated as described previously 23. Primer sequences and assay details can be found in Supporting Information Materials. For universal miRNA reverse transcription total RNA was converted to cDNA using NCode miRNA First‐Strand cDNA Synthesis Kit (Life Technologies) following the manufacturer's instructions. Real‐time PCR was performed with Invitrogen Platinum SYBR Green qPCR SuperMix (Life Technologies) and miRNA‐specific primers (Supporting Information Materials). For single miRNA‐specific analysis, RNA was reverse‐transcribed with Applied Biosystems TaqMan MicroRNA Reverse Transcription Kit (Life Technologies) and real‐time RT‐PCR performed with TaqMan MicroRNA assays (Life Technologies). All values are presented as the mean ± SEM of replicates in pooled experiments. For experiments with multiple MSC donors statistical testing was performed using a matched paired two‐tailed Student's *t* test on log‐transformed values to account for non‐normal distribution.

### Gene/miRNA Expression Profiling and Analysis

Illumina whole‐genome expression array Human HT‐12 V4 (Illumina, Saffron Walden, U.K.) was used to profile gene expression of RNA samples according to the manufacturer's protocol. For miR‐140‐5p target identification in HAC duplicate biological samples for each treatment were assessed. For MSC chondrogenesis gene expression triplicate biological samples for Days 0 and 14 were assessed, and single biological samples for intermediate time points. Raw expression data were analyzed using Agilent GeneSpring GX 11 (Agilent Technologies, Santa Clara, CA). Raw data were normalized with a quantile algorithm and the baseline was transformed to the median of all samples. Exiqon 6th generation miRCURY LNA microRNA Array (Exiqon, Vedbaek, Denmark) was used to profile miRNA expression of RNA samples according to the manufacturer's protocol. Duplicate biological samples for each time point in MSC chondrogenesis were assessed. Normalization of the quantified signals (background corrected) was performed using the global Lowess regression algorithm. Expression analysis was performed in R/bioconductor using the limma package 24. Differentially expressed gene lists were ranked according to their fold changes and compared with sets of genes that were classified according to the gene ontologies for molecular function, cellular component, and biological process. Sylamer analyses were carried out to determine the enrichment of seed matches among the genes upregulated or downregulated during MSC chondrogenesis or following experimental manipulation of miR‐140‐5p levels 25. DREM (Dynamic Regulatory Event Miner) version 2.0 is software that analyzes time course expression data and additional data on interactions of transcriptional regulators with the genes, using an Input‐Output Hidden Markov Model 26. We have extended DREM to add miRNAs as possible gene regulators using data from TargetScan, and applied the miRNA regulation data to a combined time course of both miRNA and gene expression 27. A minimum log 2‐fold‐change of 1 was applied, and genes with no regulator information were retained in the analysis. The train‐test method was used for model selection and bifurcations of gene groups (but not higher order splits) were used when fitting the model.

### RNA‐Mediated Interference and miRNA Mimic/Inhibitor Transfection in MSC and HAC

For siRNA transfection, 100 nM siRNA was transfected into 40%–50% confluent MSC using Dharmacon Dharmafect 1 lipid reagent (Thermo Fisher Scientific, St. Leon‐Rot, Germany). Dharmacon siRNA SMARTpools (Thermo Fisher Scientific) of four specific siRNA duplexes (total of 100 nM siRNA) were used to target SOX9 (M‐021507) and DICER1 (M‐003483). Depletion of gene‐specific mRNA levels was calculated by comparison of expression levels with cells transfected with 100 nM siCONTROL (nontargeting siRNA 2, cat. 001210‐02; Dharmacon). For modulation of miR‐140‐5p levels in MSC and HAC, Dharmacon miRIDIAN mimics (C‐300607), miRIDIAN hairpin inhibitors (IH‐300607), or miRCURY LNA microRNA Power Inhibitors (with phosphorothioate bonds only in the extremities to avoid toxicity) (Exiqon) were transfected by the same method as above (all 100 nM). Analysis was performed in comparison with Dharmacon miRIDIAN miRNA mimic nontargeting Control #2 (CN‐002000‐01), Sigma‐Aldrich MISSION nontargeting miRNA control 2 (HMC0003), Dharmacon miRIDIAN miRNA hairpin inhibitor nontargeting Control #2 (IN‐002005‐01), or miRCURY LNA miRNA Power Inhibitor negative control B as indicated (all 100 nM). For all experiments, cells were subject to only a single transfection, either prior to induction of MSC differentiation or for the indicated time period in MSC and HAC.

### Cloning and Plasmid Transfection in SW1353 Cells

Full length miRNA target 3′UTRs were amplified from human genomic DNA using PCR primers (Supporting Information Materials) to enable Clontech In‐Fusion HD cloning (Takara Bio Europe, Saint‐Germain‐en‐Laye, France) into the pmirGLO Dual‐Luciferase miRNA Target Expression Vector (Promega, Southampton, U.K.) following the manufacturer's instructions. Mutation of the miRNA seed‐binding sites was performed using the QuikChange II Site‐Directed Mutagenesis Kit (Agilent Technologies). All vectors and mutations were sequence verified. SW1353 chondrosarcoma cells were plated overnight in 96‐well plates at 50% confluence (18,000 cells per square centimeter). Cells were first transfected with 3′UTR luciferase constructs (10 ng) using FuGENE HD transfection reagent (Promega) for 4 hours then transfected with Dharmacon miR‐140‐5p mimic (50 nM) or miRNA mimic nontargeting Control #2 using Dharmafect 1. After 24 hours of transfection, the SW1353 cells were washed and lysed using Reporter Lysis Buffer (Promega) and firefly and renilla luciferase levels determined using the Promega Dual‐Luciferase Reporter Assay System and a GloMax 96 Microplate Luminometer (Promega). The TOPFlash construct contains T cell factor (TCF) binding upstream of a luciferase reporter gene and is widely used to assess β‐catenin‐dependent signaling 28. FOPFlash contains mutated TCF binding sites and is used as a negative control. TOPFlash and FOPFlash reporter luciferase plasmids in combination with miRNA mimic were transfected for 24 hours as above then cells were stimulated with recombinant Wnt3a (R&D Systems) for 24 hours before lysis. All values are presented as the mean ± SD of replicates in pooled experiments. Significant differences between sample groups were assessed by one‐way analysis of variance followed by the Bonferroni post hoc test for multiple comparisons or a two‐tailed Students *t* test was performed for single comparisons.

### Immunoblotting

Lysates from MSCs or SW1353 cells were prepared as described previously 23. Cytoplasmic and nuclear fractions were isolated using the NE‐PER Extraction Kit (Thermo Fisher Scientific). Lysates or fractions were immunoblotted with the following antibodies: β‐catenin and Lamin A/C (Cell Signaling Technology), and GAPDH (Millipore). Secondary anti‐rabbit antibodies were from Dako (Ely, U.K.) and chemi‐luminescent images were captured using a G:BOX Chemi system (Syngene, Cambridge, U.K.).

## Results

**Figure 1:**
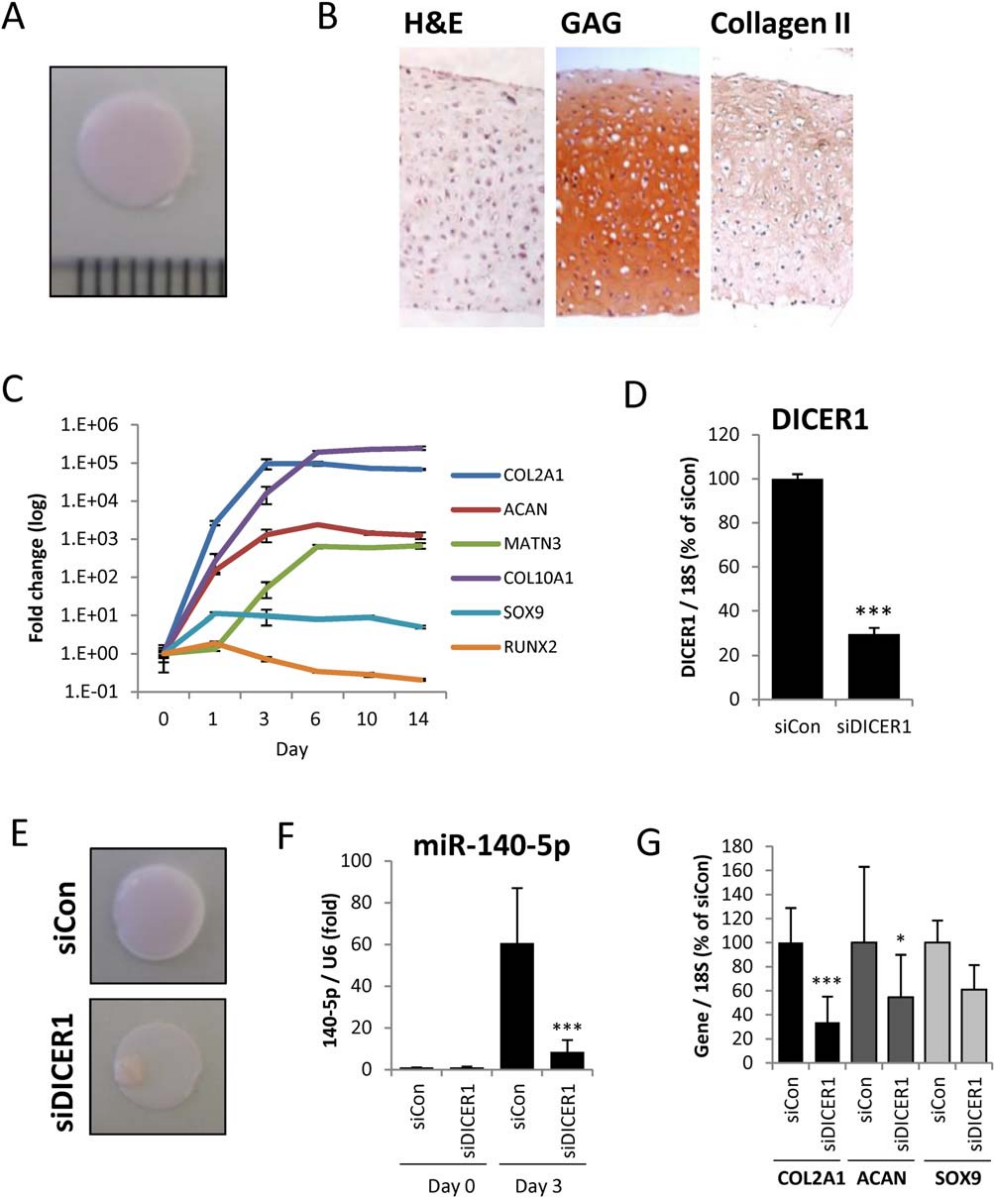
MicroRNA processing machinery is essential for chondrogenesis. **(A):** Mesenchymal stem cells (MSCs) were cultured in chondrogenic differentiation medium for 14 days in hanging transwell inserts to form a cartilaginous disc. **(B):** Day 14 chondrogenic discs were fixed, sectioned, and stained with H&E, safranin O (GAG), or anti‐collagen II antibody. **(C):** RNA was extracted from MSCs undergoing chondrogenic differentiation at the indicated time points between Day 0 and Day 14, and expression of the indicated genes measured by real‐time RT‐PCR. **(D–G):** MSCs were transfected for 3 days with DICER1‐targeting or nontargeting control siRNA prior to chondrogenic differentiation for 3 days in hanging transwell inserts. RNA was extracted at Days 0 and 3, and the indicated gene expression assessed by real‐time RT‐PCR. (D): DICER1 expression at Day 0 normalized to 18S. (E): Day 3 chondrogenic discs overlaid on transwell membrane. (F): miR‐140‐5p expression normalized to U6. (G): Day 3 chondrogenesis gene expression normalized to 18S. (A), (B), (C), and (E) are representative of experiments performed in four MSC donors. Values in (D), (F), and (G) are the mean ± SEM of data pooled from four separate MSC donors. *, *p* < .01; ***, *p* < .001 for DICER1 siRNA versus nontargeting siRNA for each gene per time point. Abbreviation: H&E, hematoxylin and eosin.

MSCs were cultured in chondrogenic differentiation medium for 14 days in Transwell hanging cell culture inserts. A flexible, translucent cartilage‐like disc is formed (Fig. 1A) which exhibits homogeneous distribution of matrix components such as sulphated GAG and type II collagen (Fig. 1B). Gene expression for a number of cartilage ECM components and transcription factors indicates a robust differentiation (Fig. 1C), as has previously been described 3. This chondrogenic differentiation is highly reproducible between different MSC donors (Supporting Information Fig. S2).

We used siRNA targeting the DICER1 gene to investigate the role of Dicer‐dependent miRNAs during chondrogenesis. Depletion of DICER1 (Fig. 1D) disrupted formation of the cartilage‐like disc early in the 14 day differentiation process (Fig. 1E). Induction of miRNAs such as miR‐140‐5p was abrogated by the loss of DICER (Fig. 1F). This was accompanied by a failure of the cells to effectively upregulate gene expression of selected cartilage components to the level of nontargeting siRNA (Fig. 1G), together indicating that miRNAs are essential for the process of chondrogenic differentiation.

**Figure 2:**
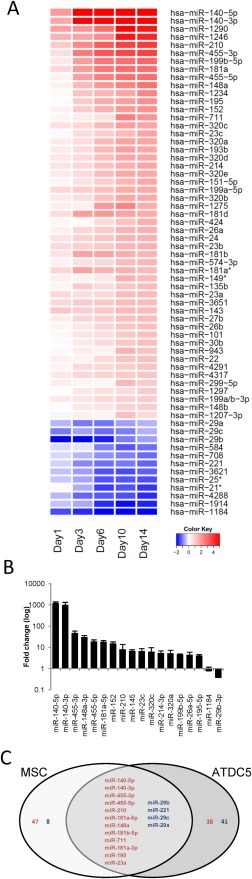
Profile of miRNA expression during chondrogenesis. MSCs were cultured in chondrogenic differentiation medium for 14 days in hanging transwell inserts to form a cartilaginous disc. RNA was extracted at the indicated time points between Day 0 and Day 14. **(A):** Heatmap representation of more than twofold significantly regulated miRNA expression assessed by microarray from one MSC donor. **(B):** Expression of selected miRNAs in data pooled three from MSC donors by real‐time RT‐PCR. **(C):** Venn diagram of common miRNAs regulated in human MSC and mouse ATDC5 chondrogenesis. miRNAs in red or blue are upregulated or downregulated, respectively, in both models of chondrogenesis. Abbreviation: MSC, mesenchymal stem cell.

In order to establish which miRNAs might regulate chondrogenesis we profiled the expression of miRNAs during MSC chondrogenic differentiation by Exiqon microarray. Of the 1,048 miRNAs measured, 51 were significantly upregulated greater than twofold during the 14 days time course, and 12 downregulated (Fig. 2A, Supporting Information Table S1). The regulation of a number of the most upregulated and downregulated miRNAs was validated by qRT‐PCR in additional MSC donors (Fig. 2B). We previously profiled miRNA expression during mouse ATDC5 chondrogenesis 12 and of the miRNAs common to both human and mouse, 12 were upregulated in both MSC and ATDC5 chondrogenesis suggesting association with the chondrocyte phenotype (Fig. 2C). Four were downregulated in both models.

**Figure 3:**
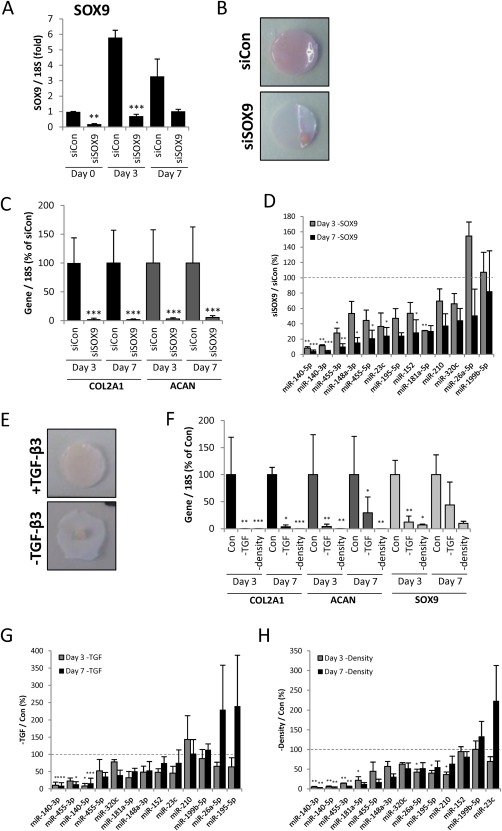
Requirement for SOX9, TGF‐β, and high density in chondrogenic miRNA expression. **(A–D):** Mesenchymal stem cells (MSCs) were transfected for 3 days with SOX9‐targeting or nontargeting control siRNA prior to chondrogenic differentiation for 7 days in hanging transwell inserts. RNA was extracted at Days 0, 3, and 7, and the indicated gene expression assessed by real‐time RT‐PCR. (A): SOX9 expression normalized to 18S. (B): Day 3 chondrogenesis disc overlaid on transwell membrane. (C): Days 3 and 7 chondrogenesis gene expression normalized to 18S. (D): Days 3 and 7 chondrogenesis miRNA expression following SOX9 depletion. Expression is normalized to U6 and presented as a percentage of nontargeting control levels. **(E–H):** MSCs were cultured in chondrogenic differentiation medium with or without TGF‐β3 for 7 days in hanging transwell inserts to form a cartilaginous disc or in monolayer at low cell density. (E): Day 3 chondrogenesis disc overlaid on transwell membrane. (F): Days 3 and 7 chondrogenesis gene expression normalized to 18S. (G, H): Days 3 and 7 chondrogenesis miRNA expression following (G) TGF‐β3 removal or (H) monolayer culture. Expression is normalized to U6 and presented as a percentage of control levels. Values are the mean ± SEM of data pooled from three separate MSC donors. *, *p* < .05; **, *p* < .01; ***, *p* < .001 for (A–D) SOX9 siRNA versus nontargeting siRNA or (E–H) the specified treatment versus control for each gene or miRNA per time point.

To further establish which miRNAs are central to chondrocyte biology we assessed their dependence on chondrogenic factors including SOX9, TGF‐β, and cell condensation. Depletion of SOX9 by siRNA (Fig. 3A) prevented formation of the cartilage‐like disc during differentiation (Fig. 3B) and upregulation of cartilage gene expression (Fig. 3C). Expression of chondrocyte‐restricted miRNAs miR‐140 and miR‐455 was markedly reduced in the absence of SOX9 (Fig. 3D). Induction of a number of other miRNAs was also diminished following depletion of SOX9, including miRs 181a‐5p, 148a‐3p, 152, and 23c. Removal of TGF‐β3 or culturing of cells at low density during differentiation also inhibits chondrogenic disc formation (Fig. 3E) and gene expression (Fig. 3F). Similarly miR‐140 and miR‐455 induction are particularly dependent on these factors and to a lesser extent a number of other miRNAs (Fig. 3G). In contrast, the expression of a number of miRNAs was independent of TGF‐β (miRs 210, 199b‐5p, 26a, and 195‐5p) or condensation (miRs 152, 199b‐5p, and 23c).

**Figure 4:**
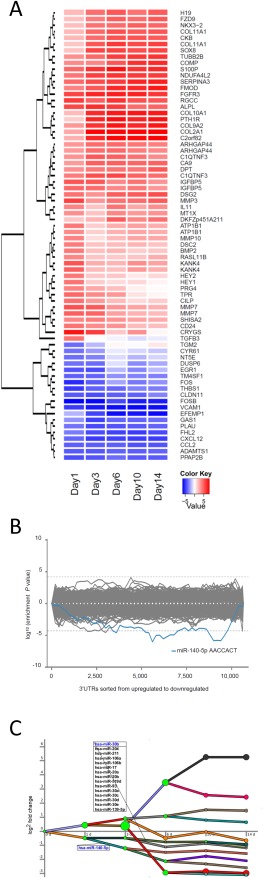
Profile of mRNA expression during chondrogenesis. Mesenchymal stem cells (MSCs) were cultured in chondrogenic differentiation medium for 14 days in hanging transwell inserts to form a cartilaginous disc. RNA was extracted at the indicated time points between Day 0 and Day 14 and assessed by microarray for one MSC donor. **(A):** Heatmap and hierarchical clustering representation of top 50 gene expression changes assessed by microarray. **(B):** Sylamer analysis performed on the sorted gene list of expression changes at Day 14 compared to Day 0. Shown are Sylamer enrichment landscape plots for seven nt sequence words. The blue line represents miR‐140‐5p seed (AACCACT) match sites. **(C):** Dynamic regulatory event miner (DREM) analysis of microarray time course data of genes and miRNAs. The lines connect the medians of groups of regulated genes. The green nodes are those nodes where a set of genes, jointly upregulated or downregulated until that time, split. The size of the node is proportional to the SD of the expression change of set of genes passing through that node. miRs shown in blue are themselves upregulated in that segment of the time course. Only the miRs with the predicted highest confidence (*p* ≤ 5 × 10^−6^) are shown.

Following our characterization of the chondrogenic miRNA repertoire we sought to identify potential targets for these miRNAs. We profiled gene expression changes in the same MSC chondrogenic differentiation time course samples by microarray and identified more than 2,000 genes that were significantly regulated greater than twofold (Fig. 4A, Supporting Information Table S2). Further to our validation of chondrocyte gene expression (Fig. 1C), examination of gene sets upregulated during chondrogenesis by global gene ontology analysis indicated significant enrichment for ECM and skeletal system development reflecting known hallmarks of chondrogenesis (Supporting Information Table S3). Sylamer is an unbiased method for detecting overrepresented miRNA targets in ranked gene expression data 25. Sylamer analysis of the chondrogenic expression data revealed significant enrichment of 7‐mer miR‐140‐5p seed sequences among the downregulated genes suggesting that miR‐140, specifically miR‐140‐5p, has the most impact of any miRNA on gene expression during chondrogenesis (Fig. 4B). Additionally, we modified DREM software to analyze time course expression data using miRNAs as possible gene regulators using data from TargetScan 3 26, 27. We found that only expression of miR‐140‐5p targets predicted by TargetScan and assessed by DREM were more likely to be downregulated during the initial phase of chondrogenesis compared with targets of unchanged miRNAs (Fig. 4C).

**Figure 5:**
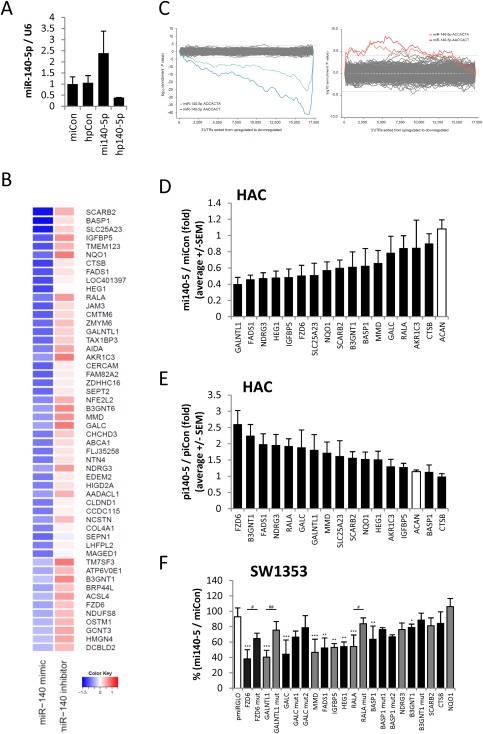
mRNA targets of miR‐140‐5p in chondrocytes. **(A–C):** HAC were transfected for 48 hours with miR‐140‐5p mimic (mi) or hairpin (hp) inhibitor, or nontargeting controls. RNA was extracted and gene expression assessed by microarray for one HAC donor. (A): miR‐140‐5p expression normalized to U6. (B): Heatmap of top 25 genes downregulated by miR‐140‐5p mimic and top 25 upregulated by miR‐140‐5p hairpin inhibitor ordered by difference between mimic and inhibitor expression levels. (C): Sylamer analysis performed on the ordered gene list of expression changes after miR‐140‐5p mimic or inhibitor treatment compared to nontargeting control. Shown are Sylamer enrichment landscape plots for seven nt sequence words. The colored lines represent miR‐140‐5p seed match sites. **(D, E):** HAC were transfected for 48 hours with miR‐140‐5p mimic (mi) or power inhibitor (pi), or nontargeting control miRNA mimic or power inhibitor. Expression of selected genes following miR‐140‐5p (D) mimic or (E) inhibitor treatment by real‐time RT‐PCR. The ACAN gene is not a target of miR‐140‐5p and therefore acted as control. Expression is normalized to 18S and presented as fold change compared to nontargeting control levels. Values are the mean ± SEM of data pooled from four separate HAC donors with each experiment performed in hextuplicate. (F): Luciferase expression in SW1353 cells following cotransfection of miR‐140‐5p target 3′UTR reporter constructs and miR‐140‐5p mimic or nontargeting control mimic for 24 hours. Expression is normalized to renilla luciferase and presented as a percentage of control levels. Values are the mean ± SD of data pooled from two to six independent experiments. *, *p* < .05; **, *p* < .01; ***, *p* < .001 for each gene‐specific 3′UTR versus empty pmiRGLO plasmid. ^#^, *p* < .05; ^##^, *p* < .01 for the indicated gene‐specific 3′UTR versus mutant gene‐specific 3′UTR. Abbreviation: HAC, human articular chondrocytes.

Our data indicate that miR‐140‐5p is the predominant miRNA strand regulating chondrogenesis. We reasoned target identification would be most successful in adult chondrocytes which express the highest levels of miR‐140‐5p (ENCODE/CSHL small RNA‐seq. and unpublished data) rather than on the dynamic transcriptome background of differentiating MSCs 29. Accordingly, we used microarray analysis to identify candidate genes regulated by miR‐140‐5p in human adult chondrocytes from articular cartilage (HAC). We over‐expressed and inhibited miR‐140‐5p (Fig. 5A) and identified genes that were upregulated or downregulated in comparison with nontargeting control treatment (Fig. 5B, Supporting Information Table S4). Sylamer analyses revealed significant enrichment of 7‐mer miR‐140‐5p seed sequences among the regulated genes (Fig. 5C). Pathway analysis highlighted that many of the miR‐140‐5p targets were found in lysosomes and membranes (Supporting Information Table S5). We selected candidate genes based on greatest repression by miR‐140‐5p mimic or greatest upregulation after miR‐140‐5p inhibition and validated many of these by real‐time qRT‐PCR in additional HAC populations (Fig. 5D, 5E). Consistent with the array, many transcripts are targeted by miR‐140‐5p with GALNTL1 the most susceptible to overexpression of miR‐140‐5p, while FZD6 and B3GNT1 were upregulated most by inhibition of miR‐140‐5p and therefore the predominant targets of endogenous miR‐140‐5p. To assess which of these candidate genes are directly targeted by miR‐140‐5p, we cloned the 3′ untranslated regions (3′UTRs) of the most regulated candidates for luciferase assays. We confirmed many as bona fide targets of miR‐140‐5p by mutation of 3′UTR target sites including FZD6, GALC, GALNTL1, MMD, and RALA (Fig. 5E).

**Figure 6:**
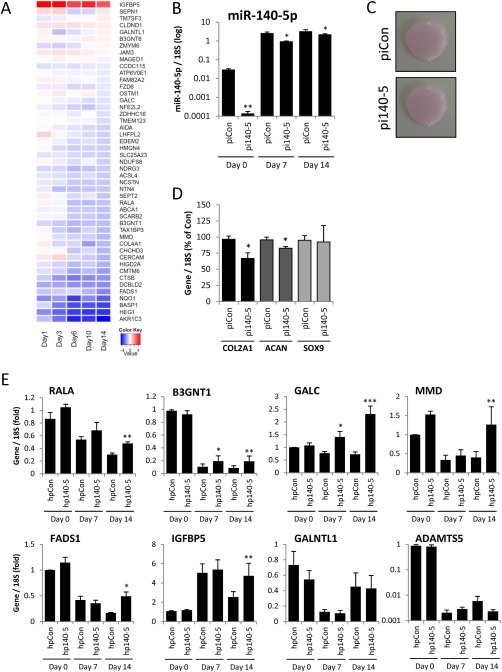
Effect of modulation of miR‐140‐5p levels on chondrogenesis, chondrocyte gene expression, and miR‐140‐5p target genes. **(A):** Heatmap of mesenchymal stem cell (MSC) chondrogenesis gene expression of the previously identified human articular chondrocytes top 50 miR‐140‐5p targets. **(B–E):** MSCs were transfected for 3 days with the indicated miR‐140‐5p inhibitors or nontargeting control inhibitors prior to chondrogenic differentiation for 14 days in hanging transwell inserts. RNA was extracted at Days 0, 7, and 14, and the indicated gene expression assessed by real‐time RT‐PCR. (B): miR‐140‐5p expression normalized to 18S. (C): Day 14 chondrogenic discs overlaid on transwell membrane. Representative of experiments performed in four MSC donors. (D): Day 14 chondrogenesis gene expression normalized to 18S. (E): Gene expression of selected experimentally verified miR‐140‐5p targets normalized to 18S. Presented as fold change compared to nontargeting control levels. Values are the mean ± SEM of data pooled from three to four separate MSC donors. *, *p* < .05; **, *p* < .01; ***, *p* < .001 for miR‐140‐5p inhibitor versus nontargeting inhibitor.

Having established the chondrocyte targets of miR‐140‐5p, we then sought to confirm these targets during the altering phenotype of MSCs undergoing chondrogenesis and increasing miR‐140‐5p levels. Examination of the expression of these miR‐140‐5p targets in our MSC chondrogenesis mRNA array indicated that the majority are downregulated during chondrocyte development (Fig. 6A). To directly examine whether miR‐140‐5p regulates these genes during chondrogenesis we inhibited miR‐140‐5p in MSCs prior to chondrogenic differentiation. Inhibition of miR‐140‐5p (Fig. 6B) had no observable effect on cartilage disc formation (Fig. 6C) or wet mass (data not shown). However, the expression of specific cartilage genes COL2A1 and ACAN was significantly reduced at Day 14 (Fig. 6D). With a pellet model of chondrogenesis we observed a modest, but not significant, reduction in cartilage GAG levels at earlier time points following miR‐140‐5p inhibition (Supporting Information Fig. S3). A number of identified targets, including RALA, B3GNT1, and GALC, were derepressed following inhibition of miR‐140‐5p indicating that their downregulation during chondrogenesis is a function of miR‐140‐5p activity (Fig. 6E). RALA has been recently implicated as a target of miR‐140 and may function in regulation of SOX9 levels 30. In contrast, GALNTL1 and ADAMTS5 expressions were not derepressed following inhibition of miR‐140‐5p.

**Figure 7:**
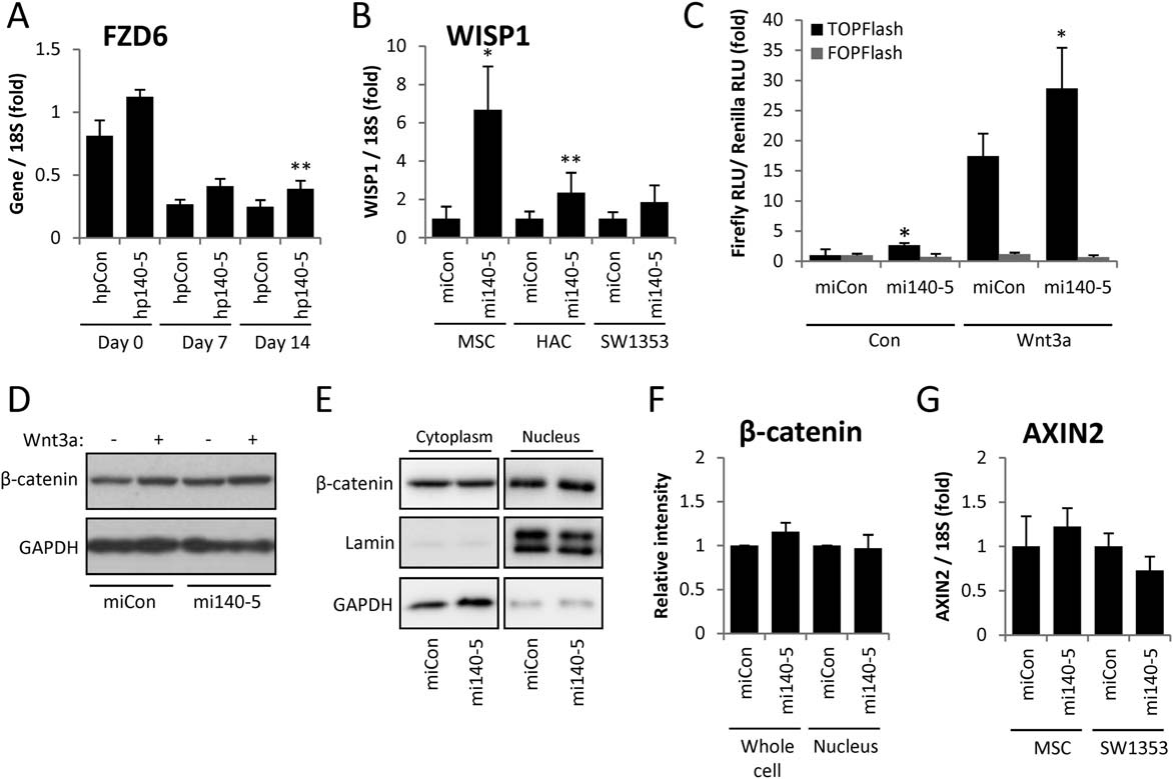
Effect of miR‐140‐5p on the Wnt signaling pathway. **(A):** MSCs were transfected for 3 days with miR‐140‐5p inhibitor or nontargeting control inhibitor prior to chondrogenic differentiation for 14 days in hanging transwell inserts. RNA was extracted at Days 0, 7, and 14, and FZD6 gene expression assessed by real‐time RT‐PCR. Presented as fold change compared to nontargeting control levels. **, *p* < .01 for miR‐140‐5p inhibitor versus nontargeting inhibitor. **(B):** MSC, human articular chondrocytes (HAC), or SW1353 cells were transfected with miR‐140‐5p mimic (mi) or nontargeting control miRNA mimic for 48 hours. WISP1 gene expression normalized to 18S. **(C):** Luciferase expression in SW1353 cells following cotransfection of TOPFlash/FOPFlash Wnt reporter constructs and miR‐140‐5p mimic or nontargeting control mimic for 24 hours +/− Wnt3a (100 ng/ml) stimulation for 24 hours. Expression is normalized to renilla luciferase and presented as fold change compared to nontargeting control levels. Values are the mean ± SD of data pooled from three independent experiments. *, *p* < .05 for miR‐140‐5p inhibitor versus nontargeting inhibitor. **(D):** MSCs were transfected with miR‐140‐5p mimic or nontargeting control miRNA mimic for 24 hours +/− Wnt3a (100 ng/ml) stimulation for 24 hours. Whole cell protein was extracted and the indicated proteins measured by immunoblotting. **(E):** SW1353 cells were transfected with miR‐140‐5p mimic or nontargeting control miRNA mimic for 24 hours. Cytoplasmic and nuclear protein fractions were extracted for immunoblotting. Anti‐Lamin and anti‐GAPDH antibodies were used to confirm nuclear and cytoplasmic fractionation, respectively. **(F):** Quantification of β‐catenin immunoblot whole cell and nuclear levels in four MSC donors and three independent SW1353 cell experiments. **(G):** AXIN2 expression after 48 hours normalized to 18S as for (B). For real‐time PCR, values are the mean ± SEM of data pooled from three to four separate MSC or HAC donors, or three independent experiments in SW1353 cells. Abbreviation: MSC, mesenchymal stem cell.

FZD6 emerged as a predominant target of miR‐140‐5p at the transcript level and in 3′UTR luciferase studies (Fig. 5E, 5G). During chondrogenesis, FZD6 is downregulated in a miR‐140‐5p‐dependent manner (Fig. 7A). FZD6 is a receptor for the Wnt signaling pathway which may direct signaling via the noncanonical pathway 31, 32. miRNAs are increasingly proposed to regulate targets in pathways and consistent with this we found additional transcripts associated with the Wnt pathway were regulated by miR‐140‐5p including AIDA, TAX1BP3, OSTM1, GPR177, and FZD9 (Fig. 5B) 33, 34, 35, 36, 37. Furthermore, miR‐140‐5p upregulated the expression of Wnt‐responsive gene Wnt1‐induced signaling protein 1 (WISP1) (Fig. 7B). Using a well‐established Wnt‐inducible luciferase reporter we found that miR‐140‐5p promoted the activation of the Wnt pathway (Fig. 7C). This was not mediated through an increase in cellular β‐catenin levels (Fig. 7D) or nuclear translocation of β‐catenin (Fig. 7E) in MSCs or SW1353 cells. In addition, there was no induction of other classic Wnt‐responsive genes such as AXIN2 (Fig. 7G).

## Discussion

With the use of a well‐defined efficient chondrogenesis model, we have presented the first integrated analysis of gene expression and miRNA expression during chondrocyte development. By modulating elements of the differentiation we have assessed the expression requirements of miRNAs during chondrogenesis to establish a fully characterized chondrocyte repertoire of miRNAs. Overwhelmingly the predominant miRNA operator during chondrogenesis was miR‐140 and for the first time we provide evidence that the miR‐140‐5p strand is likely to mediate the majority of miR‐140 effects. Consistent with other studies, we demonstrate the requirement for miR‐140‐5p in chondrocyte development but additionally we expand considerably the experimentally verified targets for miR‐140‐5p in chondrocytes and additionally confirm their regulation by miR‐140‐5p during chondrogenesis. A number of targets have links with Wnt pathways and as a result we identify a previously unappreciated role for miR‐140‐5p in promoting Wnt signaling.

Previous studies have sought to identify miRNA expression changes in models of chondrogenesis 12, 38, 39. We used an optimized model of chondrogenesis in transwells which is characterized by rapid differentiation and uniform distribution of a cartilage‐like matrix 3. This was evidenced by significant upregulation of gene expression and matrix molecule staining. Dicer, and by association miRNAs, is essential for chondrocyte proliferation and maintenance of the prehypertrophic state 9, 40. Severe skeletal defects result from loss of Dicer in chondrocytes in mice. Dicer and miRNAs appear similarly essential for our in vitro chondrogenesis system whereby its depletion results in failure of cartilage disc formation and incomplete upregulation of the chondrocyte gene expression program. The miRNA processing machinery is similarly required for differentiation of MSCs into osteoblast and adipocyte lineages suggesting that proper levels of mature miRNAs are essential for stem cell differentiation 41.

We identified a number of miRNAs regulated during chondrogenesis, the majority upregulated. Many of the upregulated miRNAs are consistent with our previous study of chondrogenesis in mouse ATDC5 cells supportive of their role during chondrogenesis. Specifically both ‐5p and ‐3p strands of cartilage‐restricted miR‐140 and miR‐455 are highly elevated during MSC chondrogenesis 10, 12. A number of other miRNAs already associated with chondrocyte function are also upregulated including miR‐148a and miR‐199 42, 43. However, comparison with miRNAs upregulated during MSC differentiation into other lineages suggests that some miRNAs are associated with differentiation per se rather than chondrogenesis specifically. For example, miRs‐23, ‐26, ‐199a, and ‐152 are also upregulated during either or both MSC osteogenesis or adipogenesis 41. miR‐1246 and miR‐1290 are upregulated at a later stage in chondrogenesis but recent evidence suggests that miR‐1246 is a pseudo‐miRNA nuclear fragment of the longer U2 small nuclear RNA 44, and miR‐1290 is almost an exact +2 frameshift sequence of miR‐1246 and assay detection may not differentiate between the two.

To establish which of the regulated miRNAs are more specific to chondrogenesis, rather than MSC differentiation, we removed critical aspects of the chondrogenic program including SOX9, TGF‐β, and high density 45. SOX9 is the master transcription factor in chondrogenesis regulating the expression of cartilage ECM genes 46. TGF‐β is required for post‐translational modifications and synergistic enhancement of chondrocyte gene expression with SOX9 47, while high cell density mimics the in vivo cellular condensation phase of chondrogenesis 45. SOX9, in combination with SOX5 and SOX6, initiates miR‐140 expression during chondrogenesis and in chondrocyte populations. Specifically the miR‐140 locus proximal promoter houses SOX9 binding sites within 3 kb of the transcription start site 13, 14. We demonstrate here that cartilage‐restricted miR‐455 also has a similar requirement for SOX9. The mature miR‐455 miRNAs originate from a hairpin encoded within intron 10 of the *COL27A1* gene 12. In contrast to the miR‐140 locus consensus sequence searches fail to identify any potential proximal SOX9 binding sites around the miR‐455 locus. Expression of *COL27A1* itself is regulated by SOX9 responsive elements in intron 1 48. The upregulation of miR‐455 parallels the kinetics of COL27A1 expression and thus it seems likely the increase in miR‐455 levels are a consequence of COL27A1 induction 12, 49. Other miRNAs have a varying requirement for SOX9 however the significant impact of loss of SOX9 on MSC chondrogenesis disrupts all gene expression at later time points. The absence of high cell density almost completely abolished the induction of matrix genes and chondrocyte‐restricted miR‐140 and miR‐455. miR‐181 also emerged as particularly dependent on these fundamental factors in chondrogenesis. miR‐181 has an established role as both effector and regulator of the TGF‐β signaling pathway 50, 51, 52, 53, 54, but previous studies indicate a role in promoting osteoblastogenesis and repressing cartilage gene expression 55, 56, 57. In contrast, the upregulation of miR‐199 is unaffected by loss of SOX9, TGF‐beta, or cell‐cell contacts, perhaps consistent with its upregulation in differentiation of MSCs into other lineages 41. Accordingly, we suggest only miR‐140 and miR‐455 as chondrogenesis‐specific miRNAs, with miR‐181 and miR‐148 also appearing intrinsically linked to the chondrogenic program.

Having characterized the chondrocyte miRNA profile we integrated analysis of chondrogenesis gene expression changes to establish the functional miRNAs during chondrocyte development. Application of an unbiased miRNA target site enrichment analysis by Sylamer revealed significant enrichment of miR‐140‐5p seed sequences above and beyond any other miRNA in the downregulated genes during chondrogenesis 25. The applicability of this analysis was verified by our modulation of miR‐140‐5p levels in HAC which identified a great enrichment of seed‐match‐containing mRNAs, consistent with the notion that functional miRNA modulation leads to regulation of direct targets. In addition, using an a priori knowledge approach with predicted miRNA binding sites from TargetScan DREM also highlighted the enrichment of miR‐140‐5p targets in genes downregulated during chondrogenesis. Taken together, these data suggest that miR‐140‐5p is the predominant miRNA acting during chondrogenesis. This is consistent with a recent study with mice with both miR‐140 deficiency and Lin28a overexpression, to inhibit let‐7 miRNAs, indicating that the Dicer null skeletal phenotype might be largely attributable to loss of these miRNAs only 58. Murine miR‐140 knockouts lack both ‐5p and ‐3p strands of miR‐140 as the whole locus is disrupted meaning the phenotype is unable to be pinned on the loss of one strand in particular 11, 16. However, in vitro studies have confirmed the majority of targets for the ‐5p strand of miR‐140 in chondrocytes and MSCs (Supporting Information Table S6). Interestingly, this apparent dominance of miR‐140‐5p occurs despite the cellular miR‐140‐3p levels exceeding those of miR‐140‐5p in both adult chondrocytes and MSC chondrogenesis (ENCODE/CSHL small RNA‐seq. and data not shown) 29. It is possible that miR‐140‐3p may significantly regulate only a few targets and thus its importance would not be recognized by target enrichment analyses such as Sylamer. However, the prevailing view of miRNA function supports the suppression of multiple targets with regulation often only twofold or less 59.

Most studies focus on the role of miRs during chondrogenesis without reference to the impact in primary chondrocytes. Our identification of the potential role of the ‐5p strand of miR‐140 prompted us to establish the targets of miR‐140‐5p in HAC, cells from the tissue exhibiting highest expression of miR‐140 in adult humans (ENCODE/CSHL small RNA‐seq.) 29. Inhibition of miR‐140‐5p allowed us to identify targets of the endogenous miRNA, the ideal method for indicating targets in vivo, while over‐expression of miR‐140‐5p suggested transcripts which might become targets if levels of the miRNA increase in disease as has previously reported for miR‐140‐5p in OA 12. Importantly, our data indicate that, owing to the very high endogenous levels of miR‐140, as little as a twofold change significantly impacts on the abundance of many target mRNAs. Luciferase assays confirmed many of these as direct targets, adding significantly to the number of already validated targets. Additionally, we have now validated that IGFBP5 is a direct target of miR‐140‐5p and repeated the findings identifying MMD and RALA as targets 17, 30, 60. Previously identified targets in mouse, for example, ADAMTS5, HDAC4, and DNPEP, appear to be species‐specific targets of miR‐140‐5p as we found no regulation in human chondrocytes 10, 11, 16. In addition, targets of miR‐140‐5p identified in other cell types or tissues do not appear to be regulated in chondrocytes, for example, mitofusin 1 and TGFBRI 61, 62, 63, 64, 65. With reference to the change in miR‐140 levels in OA, 16 of our top 50 miR‐140‐5p targets identified in HAC are altered in OA (Supporting Information Table S7) which raises the possibility that these disease‐associated alterations might be a consequence of miRNA expression changes 66, 67. A study correlating levels of miR‐140‐5p and its targets in OA patients and healthy controls would help address this question.

Strikingly, almost all our experimentally identified targets of miR‐140‐5p were downregulated during MSC chondrogenesis and inhibition of miR‐140‐5p during chondrogenesis indicated that miR‐140‐5p directly contributed to this downregulation. This suggests that miR‐140‐5p targets similar genes during chondrogenesis and in differentiated adult articular chondrocytes. Although it is clear miR‐140‐5p regulates multiple targets in seemingly disparate pathways, we were interested to note that FZD6 emerged as a major target from mRNA and 3′UTR studies. FZD6 has been identified as a negative regulator of the canonical Wnt signaling pathway by virtue of its inhibition of TCF/lymphoid enhancer‐binding factor (LEF) transcription factor transactivation 31, and may promote a Wnt4 mediated beta‐catenin‐independent pathway 32. FZD6 was downregulated during chondrogenesis and has been found upregulated during osteogenic differentiation, consistent with the antagonistic roles of SOX9 and RUNX2 in promoting their respective programs of differentiation 68. miR‐140‐5p also appears to repress osteogenesis in contrast to its role promoting chondrocyte development 69. Exploring the potential of miR‐140‐5p to regulate the Wnt pathway we observed upregulation of TCF/LEF reporter activity and induction of WISP1 expression, a Wnt‐induced gene 70. Such activation of Wnt signaling is consistent with a reduction in FZD6 levels and therefore loss of its negative regulation on canonical Wnt signaling. However, FZD6 levels correlate with beta‐catenin activation in MSCs 71 and disruption of the receptor reduces response to Wnt3a and levels of Wnts 72. Consistent with these contradictory mechanisms we see regulation of canonical reporter and target genes but not on canonical β‐catenin and AXIN gene levels. Interestingly, a β‐catenin‐independent activation of TOPFlash involving ATF2 has been reported 73. Using an RNAi approach we were unable to demonstrate a direct role for loss of FZD6 in mediating the upregulation of Wnt signaling by miR‐140‐5p (data not shown). In fact, a number of our top 50 targets of miR‐140‐5p have putative links with the Wnt signaling pathway including AIDA, TAX1BP3 (TIP1), OSTM1, GPR177, and FZD9 33, 34, 35, 36, 37. OSTM1 has already been demonstrated to be a direct target of miR‐140‐5p but its role in Wnt signaling in this report was not explored 74. Thus, our data suggest that miR‐140‐5p may regulate Wnt signaling via one of these identified targets.

The role of Wnt signaling in chondrocytes and skeletal development is complex and sometimes contradictory 75. Overexpression studies have indicated that Wnt3a and a number of other Wnt family members inhibit chondrogenesis from the condensation phase through a β‐catenin‐dependent mechanism 75, 76. The role of β‐catenin then switches after chondrocyte development and activation is required for the later hypertrophic maturation of chondrocytes. However, a couple of studies contradict these and suggest that Wnt3a, β‐catenin, and LEF may promote SOX9 expression and as a result chondrogenesis 77. Our data suggest that miR‐140‐5p might be able to activate TCF/LEF‐dependent gene expression while avoiding the negative effects of activating β‐catenin during chondrogenesis. miR‐140‐5p also upregulated expression of WISP1, a member of the CCN protein family, which is expressed in limb development, specifically in condensing mesenchyme, during chondrocyte terminal differentiation and in bone formation sites 70. Interestingly, WISP1 promotes proliferation of MSCs and prechondrocytes and miR‐140‐5p has previously been posited to contribute to chondrocyte proliferation 58, 70. Our pathway analysis on genes upregulated following overexpression of miR‐140‐5p indicated cell cycle regulation by miR‐140‐5p, and the upregulation of WISP1 and the activation of Wnt signaling, which has an established role in cell cycle progression and cell proliferation, also contribute to a potential for miR‐140 to promote MSC and chondrocyte proliferation.

We also over‐expressed miR‐140‐5p in MSCs prior to chondrogenic differentiation in order to address the question of whether increased miR‐140‐5p levels could enhance chondrocyte development. Contrary to expectation, the over‐expression of miR‐140‐5p impaired cartilage formation and chondrocyte gene expression (Supporting Information Fig. S4). Such immediate over‐expression may have inhibited the early stages of chondrogenesis where MSCs usually experience a more controlled increase in miR‐140‐5p levels to repress unwanted targets during differentiation. Similarly we used an RNAi approach to repress miR‐140‐5p targets RALA and FZD6 prior to chondrogenesis to determine whether their depletion would enhance chondrogenesis. Again, we found that this approach unexpectedly reduced chondrocyte differentiation (data not shown). As a result we would suggest that for engineering approaches seeking to improve chondrocyte differentiation and cartilage formation an inducible mechanism of over‐expression of miRNAs or repression of targets would be more appropriate in order to mimic the natural order of events.

Taken together, our data confirm that miR‐140‐5p is an essential regulator of chondrocyte gene expression, and establish that miR‐140‐5p actually regulates multiple targets to elicit its effect in chondrocyte development and function, including contributing to the impact of the Wnt signaling pathway. Owing to the regulation of these targets in both MSCs and HAC, the significant induction of miR‐140 during MSC chondrogenesis, and it being the most abundant miRNA in adult chondrocytes, we believe miR‐140‐5p has an equally important role in both regulating targets during MSC chondrogenesis and then maintaining the suppression of these targets in adult chondrocytes (HAC) to maintain the chondrocyte phenotype.

## Conclusions

We have characterized the miRNAs regulated during chondrogenesis and highlighted those most specific to the chondrocyte phenotype. Integrating miRNA and mRNA expression data allowed us to identify the ‐5p strand of miR‐140 as the primary miRNA regulating gene expression during chondrogenesis. Consistent with other miRNAs we have validated multiple targets of miR‐140‐5p, and in particular we identify its regulation of Wnt signaling, an essential pathway involved in skeletal development.

## Author Contributions

M.J.B.: conception and design, collection and/or assembly of data, data analysis and interpretation, and manuscript writing; M.T., R.G., and S.W.: collection and/or assembly of data and data analysis and interpretation; W.H.: collection and/or assembly of data; G.R.S. and D.P.S.: data analysis and interpretation; I.M.C.: conception and design, financial support, and manuscript writing; D.A.Y.: conception and design, financial support, data analysis and interpretation, and manuscript writing.

## Disclosure of Potential Conflicts of Interest

The authors indicate no potential conflicts of interest.

## Supporting information

Supplementary Information Abstract

Supplementary Information Figure S1

Supplementary Information Figure S2

Supplementary Information Figure S3

Supplementary Information Figure S4

Supplementary Information

Supplementary Information Table S1

Supplementary Information Table S2

Supplementary Information Table S3

Supplementary Information Table S4

Supplementary Information Table S5

Supplementary Information Table S6

Supplementary Information Table S7
