# Supplementary figures and images for: Genome‐Wide MicroRNA and Gene Analysis of Mesenchymal Stem Cell Chondrogenesis Identifies an Essential Role and Multiple Targets for miR‐140‐5p
Source: Stem Cells. 2015 Jul 29;33(11):3266–80. doi: 10.1002/stem.2093 (PMC4737122; doi:10.1002/stem.2093)

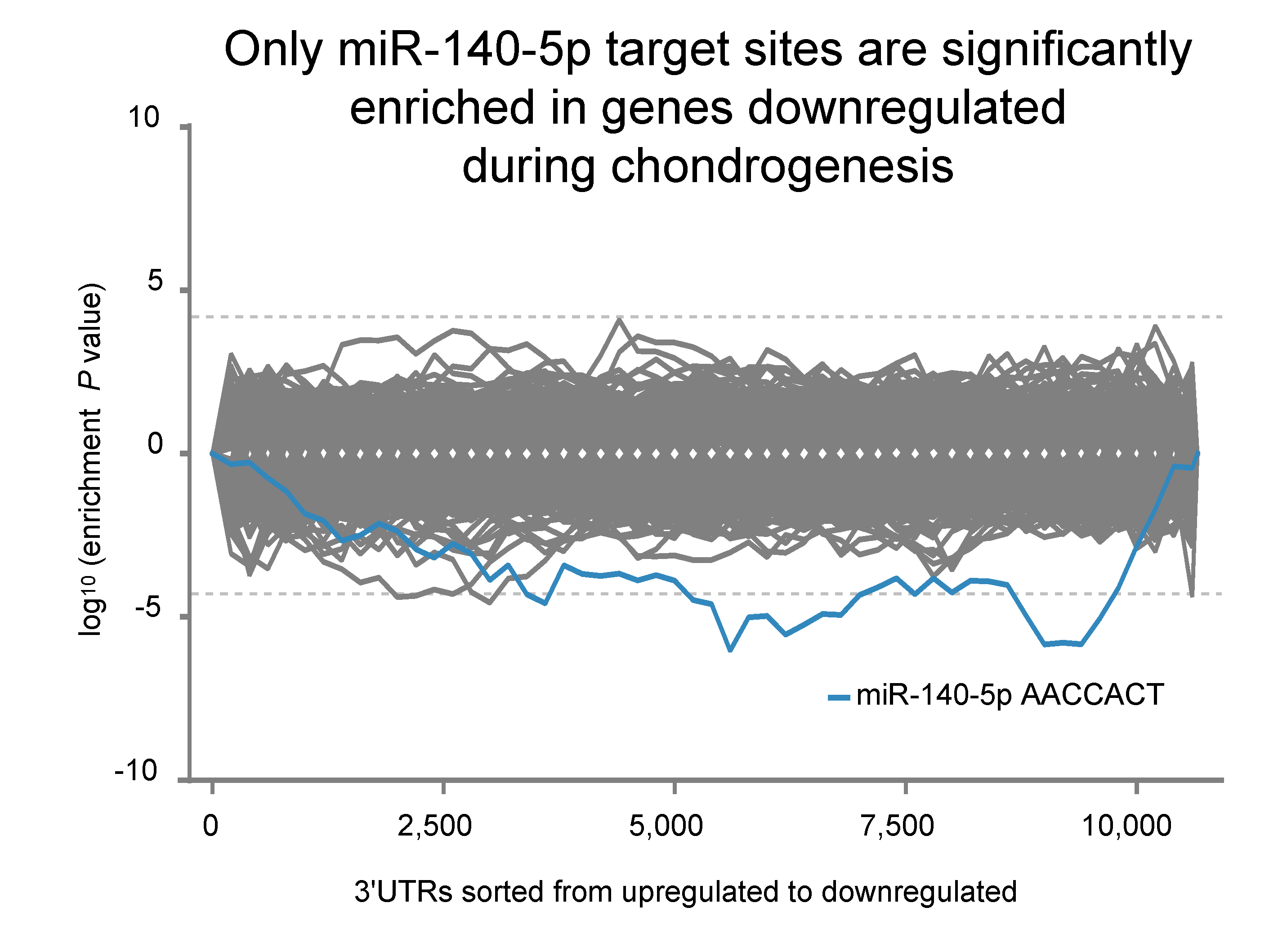

Supplement: Supplementary file 1 — Supplementary Information Abstract [file STEM-33-3266-s001.tif]

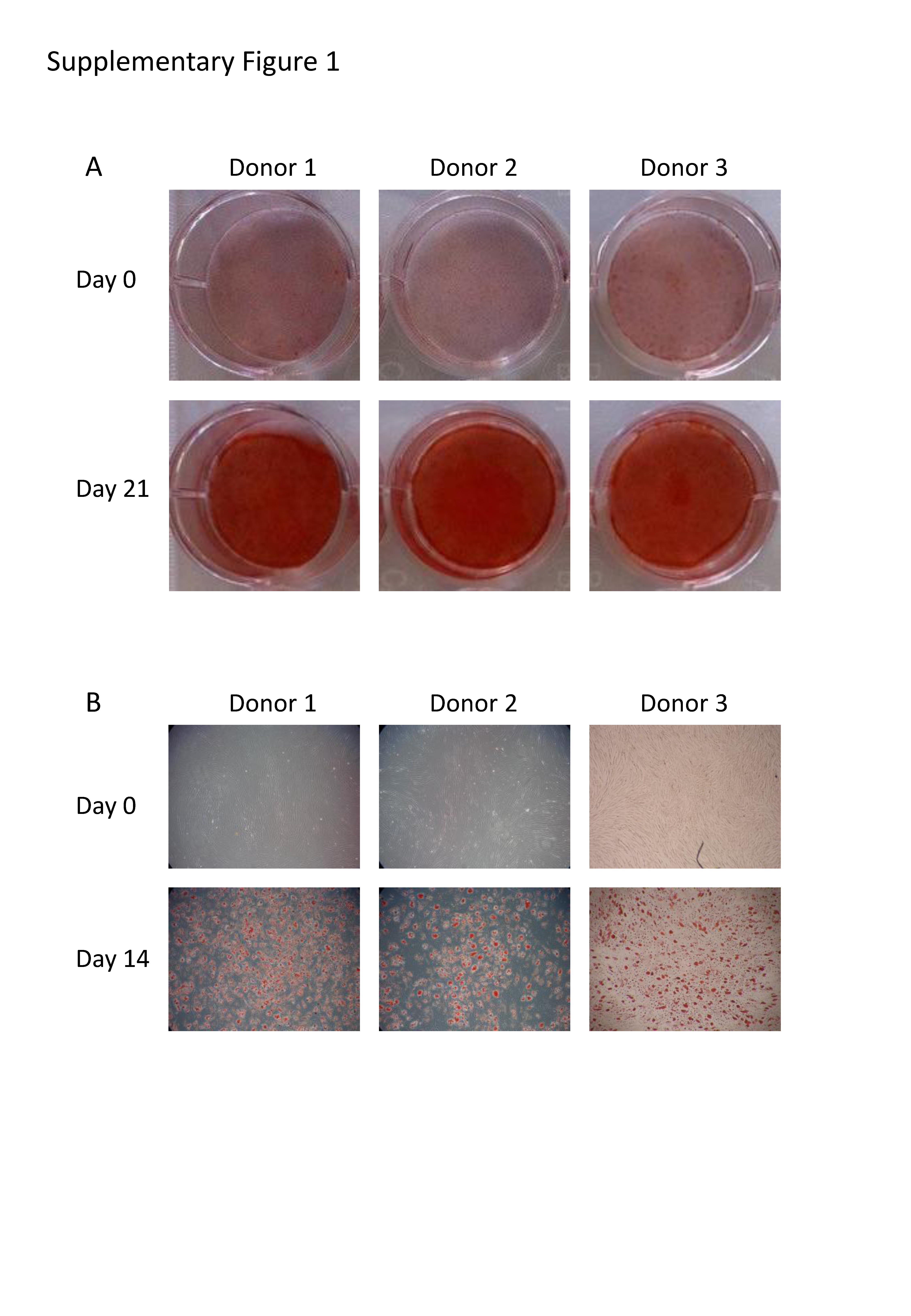

Supplement: Supplementary file 2 — Supplementary Information Figure S1 [file STEM-33-3266-s002.tif]

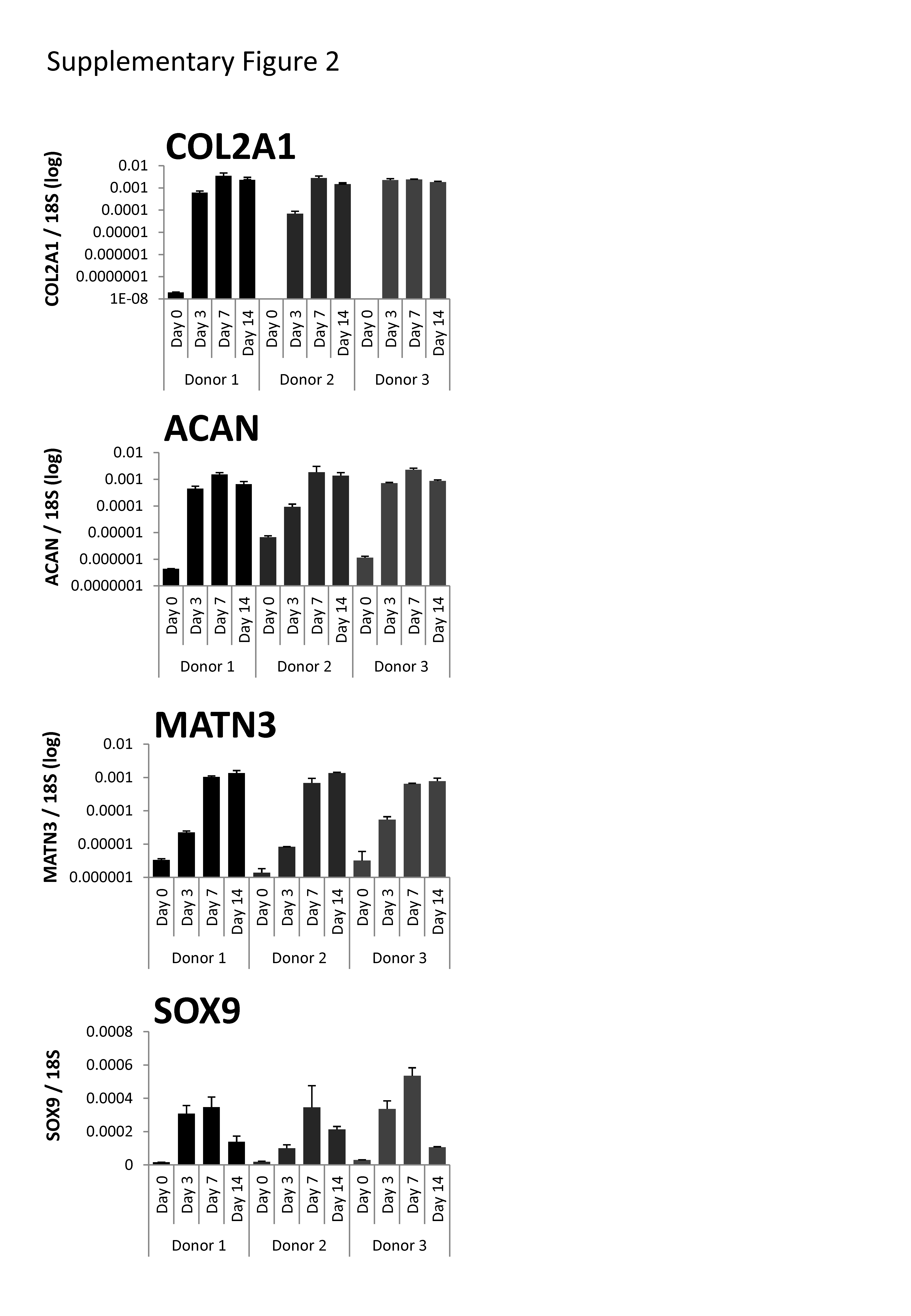

Supplement: Supplementary file 3 — Supplementary Information Figure S2 [file STEM-33-3266-s003.tif]

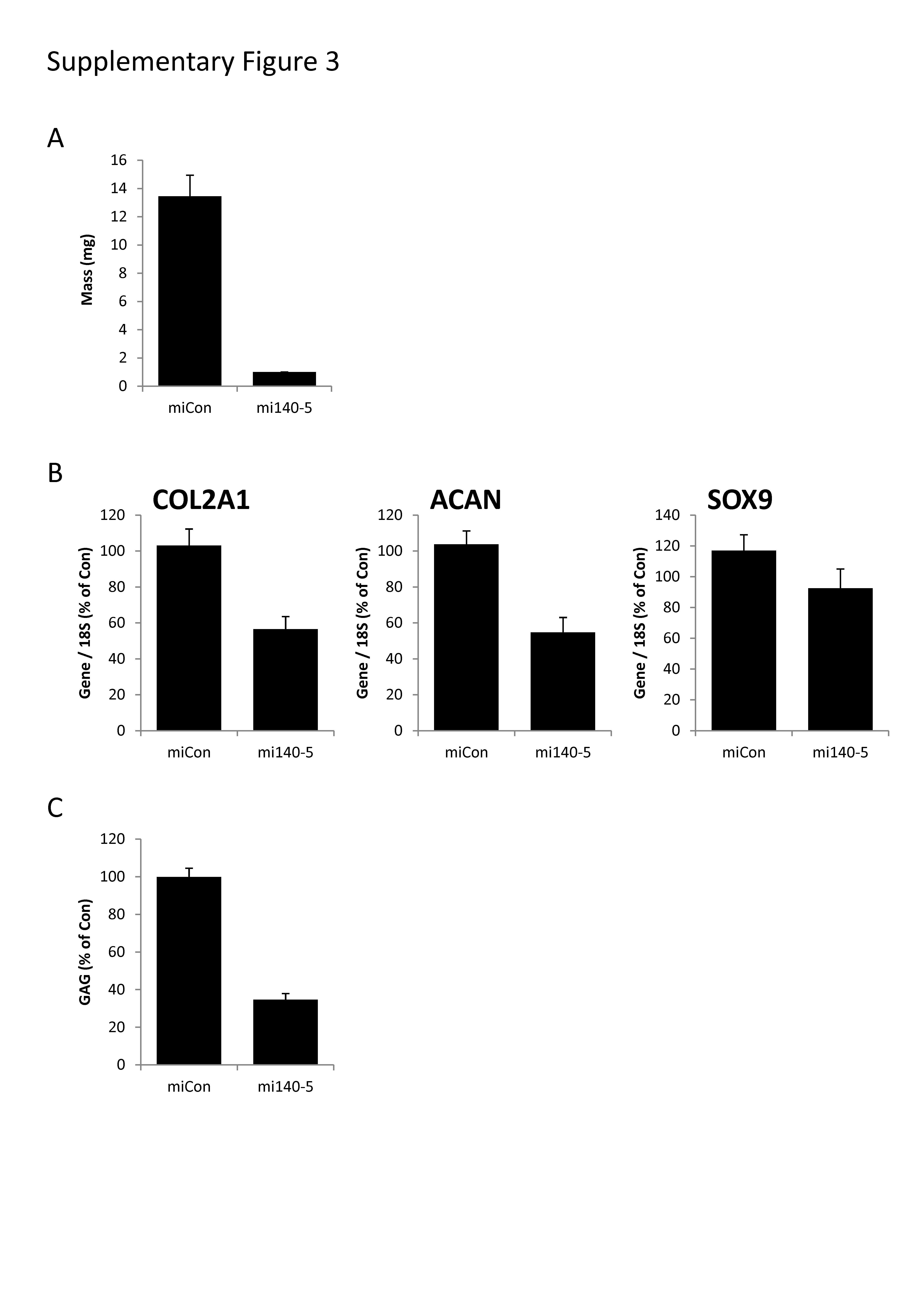

Supplement: Supplementary file 4 — Supplementary Information Figure S3 [file STEM-33-3266-s004.tif]

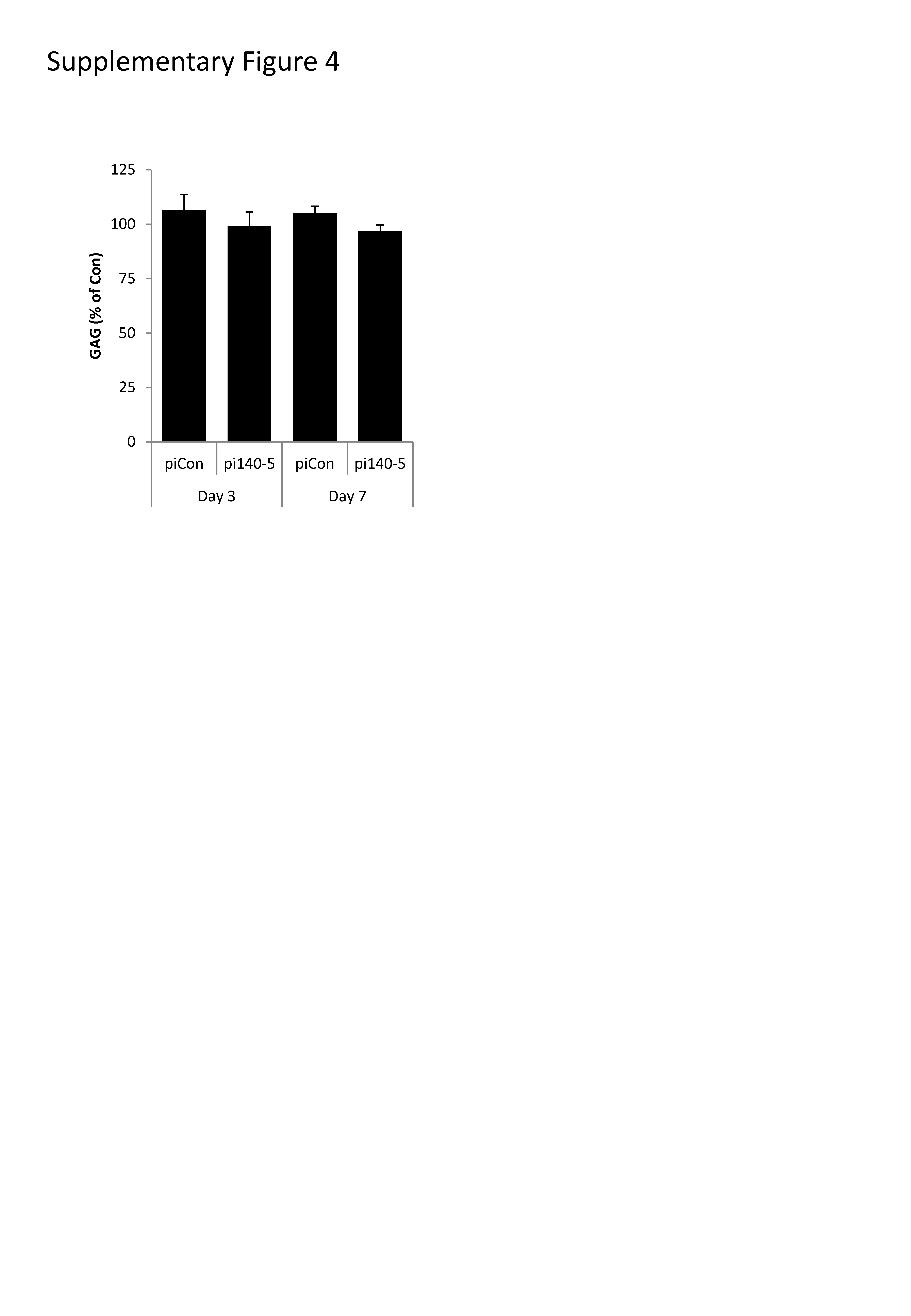

Supplement: Supplementary file 5 — Supplementary Information Figure S4 [file STEM-33-3266-s005.tif]
