# Supplementary material for: Genome‐Wide MicroRNA and Gene Analysis of Mesenchymal Stem Cell Chondrogenesis Identifies an Essential Role and Multiple Targets for miR‐140‐5p
Source: Stem Cells. 2015 Jul 29;33(11):3266–80. doi: 10.1002/stem.2093 (PMC4737122; doi:10.1002/stem.2093)
Supplement: Supplementary file 6 — Supplementary Information [file STEM-33-3266-s006.docx]

Supplementary Methods

*Osteoblast and adipocyte differentiation*

MSC were plated at a density of 15000/cm^2^ for 24 hours then media were replaced with osteoblastogenic culture medium consisting of DMEM supplemented with 10% FBS (foetal bovine serum), β-Glycerol Phosphate (5 mM), dexamethasone (10 nM) and ascorbic acid 2-phosphate (50 mg/ml) (all Sigma). Media were replaced every 3/4 days. To achieve fully mineralized cultures the cells were cultured for 21 days. To induce adipocyte differentiation the media were replaced with adipogenic induction culture medium consisting of DMEM supplemented with 10% FBS, dexamethasone (1μM), insulin (10 μg/ml), IBMX (0.5 mM), indomethacin (60μM), rosiglitazone (2 μM) and IGF-1 (20 nM; R&D Systems) (all Sigma unless specified). After 3 days, the adipogenic induction medium was replaced by the maintenance medium, which consists of DMEM supplemented with 10% FBS and insulin (10 μg/ml). After 4 days with this media the cycle of induction/maintenance ended. Two cycles were required to achieve fully developed adipocytes.

*‘Mini-pellet’ chondrogenic differentiation*

For V-bottom 96-well plate ‘mini-pellet’ chondrogenesis 5x10^4^ MSC in 150µl chondrogenic differentiation medium were pipetted into a UV-sterilised V-bottom 96-well plate and centrifuged at 500g for 5 minutes. Media were replaced every 2 or 3 days up to 7 days.

*‘Mini-pellet’ RNA extraction and real-time reverse transcription PCR*

MSC chondrogenesis mini-pellets were disrupted in Ambion Cells-to-cDNA II Cell Lysis buffer (Life Technologies). Total RNA was then extracted and converted to cDNA using MMLV reverse transcriptase (Invitrogen) and TaqMan real-time RT-PCR was performed and gene expression levels were calculated as described previously [^1^](#_ENREF_1).

Supplementary Materials

MSC donor additional information

| MSC donor | Age | Sex | Race |
| --- | --- | --- | --- |
| 1 | 22 | F | Black |
| 2 | 22 | M | Black |
| 3 | 24 | F | Black |
| 4 | 24 | F | Caucasian |
| 5 | 19 | F | Black |
| 6 | 19 | M | Black |
| 7 | 22 | M | Not reported |

HAC donor additional information

| Patient cartilage | Age | Sex | Joint |
| --- | --- | --- | --- |
| 1 | 84 | M | Hip |
| 2 | 82 | F | Hip |
| 3 | 58 | F | Hip |
| 4 | 58 | M | Knee |
| 5 | 60 | F | Knee |
| 6 | 71 | F | Knee |
| 7 | 65 | M | Knee |
| 8 | 73 | F | Knee |
| 9 | 80 | M | Knee |

IDT PrimeTime® Pre-designed qPCR Assays

| COL2A1 |
| --- |
| COL10A1 |
| SOX9 |

Roche probe library primers

| Gene | Primer | Primer sequence |
| --- | --- | --- |
| ACAN | F | agacggcttccaccagtgt |
|  | R | gggagtgtggatggggtat |
| MATN3 | F | ttccaggaaaccttctgtgc |
|  | R | tgtatccttggctacactcacagt |
| RUNX2 | F | GGAGTGGACGAGGCAAGAGTTT |
|  | R | AGCTTCTGTCTGTGCCTTCTGG |
| DICER1 | F | tgttccaggaagaccaggtt |
|  | R | actatccctcaaacactctggaa |
| WWP2 (transcript variant 2) | F | agtgcttcgactgaccatga |
|  | R | cacccgtccattgtcctg |
| COL27A1 | F | ggtcctatccaattgcaacaa |
|  | R | agccggtctggatagctgta |
| GALNTL1 | F | ccctagagaaggcaagcaga |
|  | R | gaggccactgactgaatgct |
| FADS1 | F | catcaacatgcatcccttctt |
|  | R | ttttcttctgtttcccaagctc |
| NDRG3 | F | gctgtctgtcatgtggatgc |
|  | R | catccattgtggggtactga |
| HEG1 | F | tcccagagtggcaacttagc |
|  | R | gcaattctttcatcgaactctga |
| IGFBP5 | F | ctaccgcgagcaagtcaag |
|  | R | gtctcctcggccatctca |
| FZD6 | F | gaagcaaaaagacatgcacaga |
|  | R | ttcgactttcactgattggatct |
| SLC25A23 | F | ggccagatagccagttaccc |
|  | R | tagcagacccagcatggac |
| NQO1 | F | cagctcaccgagagcctagt |
|  | R | gagtgagccagtacgatcagtg |
| SCARB2 | F | cagttctatttcttcaatgtcacca |
|  | R | aatatttgctttgtttctgagttcc |
| B3GNT1 | F | cccctgggagccattcta |
|  | R | tcaggacctcaaaatcaaacc |
| BASP1 | F | catcgtttgtacctgaaactgc |
|  | R | cccattgcagaagaaaagcta |
| MMD | F | tctttttctatctcacaatgggatt |
|  | R | ctgaagtccatcggtgttgtt |
| GALC | F | gtacgtgctcgacgactcc |
|  | R | ttactagaagtcgggaggttgc |
| RALA | F | ggaggggttcctctgtgttt |
|  | R | tggaacattctcatcttcttttactc |
| AKR1C3 | F | cattggggtgtcaaacttca |
|  | R | ccggttgaaatacggatgac |
| CTSB | F | ctgtggcagcatgtgtgg |
|  | R | gcaccctacatgggattcat |
| ADAMTS5 | F | TGTCCTGCCAGCGGATGT |
|  | R | ACGGAATTACTGTACGGCCTACA |
| WISP1 | F | ctggcagcagtgacagca |
|  | R | ggagctggggtaaagtccat |
| AXIN2 | F | ccacacccttctccaatcc |
|  | R | tgccagtttctttggctctt |

TaqMan® MicroRNA and U6 assays

| miR-140-5p |
| --- |
| miR-455-3p |
| U6 |

Ncode miRNA and U6 specific primers

| Hsa-miR-1184 | CAGCGACTTGATGGCTTCC |
| --- | --- |
| Hsa-miR-140-3p | TACCACAGGGTAGAACCACGG |
| Hsa-miR-140-5p | GCAGTGGTTTTACCCTATGGTAG |
| Hsa-miR-148a-3p | GCTCAGTGCACTACAGAACTTTGT |
| Hsa-miR-152 | TCAGTGCATGACAGAACTTGG |
| Hsa-miR-181a-5p | ATTCAACGCTGTCGGTGAGT |
| Hsa-miR-195-5p | GTAGCAGCACAGAAATATTGGC |
| Hsa-miR-199b-5p | GCCCAGTGTTTAGACTATCTGTTC |
| Hsa-miR-210 | GCGTGTGACAGCGGCTGA |
| Hsa-miR-214-3p | CAGGCACAGACAGGCAGT |
| Hsa-miR-23c | ATCACATTGCCAGTGATTACCC |
| Hsa-miR-26a-5p | GGTTCAAGTAATCCAGGATAGGCT |
| Hsa-miR-29b-3p | TAGCACCATTTGAAATCAGTGTT |
| Hsa-miR-320a | GCTGGGTTGAGAGGGCGA |
| Hsa-miR-320c | AAAAGCTGGGTTGAGAGGGT |
| Hsa-miR-455-3p | GCAGTCCATGGGCATATACAC |
| Hsa-miR-455-5p | TATGTGCCTTTGGACTACATCG |
| RNU6-2 | CGCAAGGATGACACGCAAA |

3'UTR cloning primers

| RALA 3UTR IFC F | GCTCGCTAGCCTCGACAAAGCCCAAACTCCTTTCTT |
| --- | --- |
| RALA 3UTR IFC R | CGACTCTAGACTCGAGGAAGTTTATTGGTCTTTAAATGG |
| B3GNT1 3UTR IFC F | GCTCGCTAGCCTCGACCTTCCCTCCCCTAATCTGA |
| B3GNT1 3UTR IFC R | CGACTCTAGACTCGATGGGTGTGAACACGCATTTA |
| FZD6 3UTR IFC F | GCTCGCTAGCCTCGAAGAACATTTTCTCTCGTTACTCAGAA |
| FZD6 3UTR IFC R | CGACTCTAGACTCGAATTCAAAGTTGTCTGTTTAAAATGTGG |
| NQO1 3UTR IFC F | GCTCGCTAGCCTCGATTCCTTAGCCTGGATTTCCTT |
| NQO1 3UTR IFC R | CGACTCTAGACTCGATTGAGAAATTTACACAAAATTGCAG |
| GALNTL1 3UTR IFC F | GCTCGCTAGCCTCGACGGAGGGATTTTCTTGAAGG |
| GALNTL1 3UTR IFC R | CGACTCTAGACTCGAGACATGTAAGAAGTGGTTTTATTCCA |
| SCARB2 3UTR IFC F | GCTCGCTAGCCTCGAACATTGCCTTTGCTTGGTG |
| SCARB2 3UTR IFC R | CGACTCTAGACTCGAATAAAGTGGTTTTTTATTAA |
| BASP1 3UTR IFC F | GCTCGCTAGCCTCGACAAGGACAGCCTATAGGAAAAA |
| BASP1 3UTR IFC R | CGACTCTAGACTCGATACTGTTGCACCATTTATACAATTACA |
| GALC 3UTR IFC F | GCTCGCTAGCCTCGAGGGCATCATAGAATACTCTGGATTT |
| GALC 3UTR IFC R | CGACTCTAGACTCGATTTTAATTGTAGTATCAGAAACTGGTG |
| MMD 3UTR IFC F | GCTCGCTAGCCTCGACCAATCTGTACTAATTCTCCAAACC |
| MMD 3UTR IFC R | CGACTCTAGACTCGATGGAACAAATCCAAGTAACAACTTT |
| CTSB 3UTR IFC F | GCTCGCTAGCCTCGAGCCTGTCGTGCCAGTCCT |
| CTSB 3UTR IFC R | CGACTCTAGACTCGATTGGGAGCAGGGAGAACTTT |
| HEG1 3UTR IFC F | GCTCGCTAGCCTCGAATTGCTCTGAGCCAGTCACC |
| HEG1 3UTR IFC R | CGACTCTAGACTCGAGGGCCATTGGTTCTACAGAG |
| FADS1 3UTR IFC F | GCTCGCTAGCCTCGATGCCCAGTCTGGAAGAAGAG |
| FADS1 3UTR IFC R | CGACTCTAGACTCGATTAACTTTATTTTTCAACTTCATAGGG |
| IGFBP5 3UTR IFC F | GCTCGCTAGCCTCGATGTCCCCTAAACCCAACTCA |
| IGFBP5 3UTR IFC R | CGACTCTAGACTCGACATGACAAAAGACAAAGATCAAGG |
| NDRG3 3UTR IFC F | GCTCGCTAGCCTCGAAGATGCTCCTCCCCTGGAC |
| NDRG3 3UTR IFC R | CGACTCTAGACTCGACAGGTAACAATGAGACATATGCAG |

3'UTR mutation primers

| RALA QC mut F | ttaaccaggggtccttagctcaaacattgtgactttg |
| --- | --- |
| RALA QC mut R | caaagtcacaatgtttgagctaaggacccctggttaa |
| B3GNT1 QC mut F | gatcgattcctagctttagctcaaactagctgtgtgg |
| B3GNT1 QC mut R | ccacacagctagtttgagctaaagctaggaatcgatc |
| GALNTL1 QC mut F | acacacctggaataatagctcatcttacatgtctcga |
| GALNTL1 QC mut R | tcgagacatgtaagatgagctattattccaggtgtgt |
| FZD6 QC mut F | tgattgtattataacgcgttacagttgcttata |
| FZD6 QC mut R | tataagcaactgtaacgcgttataatacaatca |
| GALC QC mut1 F | cataacaggttgaacgcgttttcaaaaagctac |
| GALC QC mut1 R | gtagctttttgaaaacgcgttcaacctgttatg |
| GALC QC mut2 F | cacagagagttatacgcgttacaagaccaaaac |
| GALC QC mut2 R | gttttggtcttgtaacgcgtataactctctgtg |
| BASP1 QC mut1 F | ggagattgttttaacgcgtattgtttttcctat |
| BASP1 QC mut1 R | ataggaaaaacaatacgcgttaaaacaatctcc |
| BASP1 QC mut2 F | cagacaccaacacacgcgtcattggaaaatgga |
| BASP1 QC mut2 R | tccattttccaatgacgcgtgtgttggtgtctg |

1. Barter MJ, Hui W, Lakey RL, et al. Lipophilic statins prevent matrix metalloproteinase-mediated cartilage collagen breakdown by inhibiting protein geranylgeranylation. Annals of the rheumatic diseases*.* 2010;69:2189-2198.
